# Supplementary material for: Energy systems in scenarios at net-zero CO2 emissions
Source: Nat Commun. 2021 Oct 20;12:6096. doi: 10.1038/s41467-021-26356-y (PMC8528892; doi:10.1038/s41467-021-26356-y)
Supplement: Supplementary file 1 — Supplementary Information [file 41467_2021_26356_MOESM1_ESM.pdf]

Supplementary Information for

Energy systems in scenarios at net-zero CO_2 emissions

DeAngelo et al.

Supplementary Table 1 | List of 177 scenarios evaluated in this study that reach global net-zero CO_2 emissions and included all output variables required for our analysis. Scenarios are listed first, followed by the model that ran the scenario (format: scenario, model).

1	ADVANCE_2020_1.5C-2100, AIM/CGE 2.0
2	ADVANCE_2020_1.5C-2100, IMAGE 3.0.1
3	ADVANCE_2020_1.5C-2100, MESSAGE-GLOBIOM 1.0
4	ADVANCE_2020_1.5C-2100, POLES ADVANCE
5	ADVANCE_2020_1.5C-2100, REMIND 1.7
6	ADVANCE_2020_1.5C-2100, WITCH-GLOBIOM 4.2
7	ADVANCE_2020_Med2C, MESSAGE-GLOBIOM 1.0
8	ADVANCE_2020_Med2C, POLES ADVANCE
9	ADVANCE_2020_Med2C, REMIND 1.7
10	ADVANCE_2020_Med2C, WITCH-GLOBIOM 4.2
11	ADVANCE_2020_WB2C, AIM/CGE 2.0
12	ADVANCE_2020_WB2C, IMAGE 3.0.1
13	ADVANCE_2020_WB2C, MESSAGE-GLOBIOM 1.0
14	ADVANCE_2020_WB2C, POLES ADVANCE
15	ADVANCE_2020_WB2C, REMIND 1.7
16	ADVANCE_2020_WB2C, WITCH-GLOBIOM 4.2
17	ADVANCE_2030_1.5C-2100, POLES ADVANCE
18	ADVANCE_2030_1.5C-2100, REMIND 1.7
19	ADVANCE_2030_Med2C, IMAGE 3.0.1
20	ADVANCE_2030_Med2C, MESSAGE-GLOBIOM 1.0
21	ADVANCE_2030_Med2C, POLES ADVANCE
22	ADVANCE_2030_Med2C, REMIND 1.7
23	ADVANCE_2030_Med2C, WITCH-GLOBIOM 4.2
24	ADVANCE_2030_Price1.5C, AIM/CGE 2.0
25	ADVANCE_2030_Price1.5C, MESSAGE-GLOBIOM 1.0
26	ADVANCE_2030_Price1.5C, POLES ADVANCE
27	ADVANCE_2030_Price1.5C, REMIND 1.7
28	ADVANCE_2030_Price1.5C, WITCH-GLOBIOM 4.2
29	ADVANCE_2030_WB2C, AIM/CGE 2.0

30	ADVANCE_2030_WB2C, IMAGE 3.0.1
31	ADVANCE_2030_WB2C, MESSAGE-GLOBIOM 1.0
32	ADVANCE_2030_WB2C, POLES ADVANCE
33	ADVANCE_2030_WB2C, REMIND 1.7
34	ADVANCE_2030_WB2C, WITCH-GLOBIOM 4.2
35	CD-LINKS_NPi2020_1000, IMAGE 3.0.1
36	CD-LINKS_NPi2020_1000, MESSAGEix-GLOBIOM 1.0
37	CD-LINKS_NPi2020_1000, POLES CD-LINKS
38	CD-LINKS_NPi2020_1000, REMIND-MAgPIE 1.7-3.0
39	CD-LINKS_NPi2020_1000, WITCH-GLOBIOM 4.4
40	CD-LINKS_NPi2020_1600, IMAGE 3.0.1
41	CD-LINKS_NPi2020_1600, MESSAGEix-GLOBIOM 1.0
42	CD-LINKS_NPi2020_1600, POLES CD-LINKS
43	CD-LINKS_NPi2020_1600, REMIND-MAgPIE 1.7-3.0
44	CD-LINKS_NPi2020_1600, WITCH-GLOBIOM 4.4
45	CD-LINKS_NPi2020_400, AIM/CGE 2.1
46	CD-LINKS_NPi2020_400, IMAGE 3.0.1
47	CD-LINKS_NPi2020_400, MESSAGEix-GLOBIOM 1.0
48	CD-LINKS_NPi2020_400, POLES CD-LINKS
49	CD-LINKS_NPi2020_400, REMIND-MAgPIE 1.7-3.0
50	CD-LINKS_NPi2020_400, WITCH-GLOBIOM 4.4
51	CEMICS-1.5-CDR12, REMIND 1.7
52	CEMICS-1.5-CDR20, REMIND 1.7
53	CEMICS-1.5-CDR8, REMIND 1.7
54	CEMICS-2.0-CDR12, REMIND 1.7
55	CEMICS-2.0-CDR20, REMIND 1.7
56	CEMICS-2.0-CDR8, REMIND 1.7
57	EMF33_1.5C_cost100, MESSAGE-GLOBIOM 1.0
58	EMF33_1.5C_cost100, POLES EMF33
59	EMF33_1.5C_cost100, REMIND-MAgPIE 1.7-3.0
60	EMF33_1.5C_full, MESSAGE-GLOBIOM 1.0
61	EMF33_1.5C_full, POLES EMF33
62	EMF33_1.5C_full, REMIND-MAgPIE 1.7-3.0
63	EMF33_1.5C_limbio, POLES EMF33
64	EMF33_1.5C_nofuel, POLES EMF33
65	EMF33_1.5C_nofuel, REMIND-MAgPIE 1.7-3.0
66	EMF33_Med2C_cost100, MESSAGE-GLOBIOM 1.0

67	EMF33_Med2C_cost100, POLES EMF33
68	EMF33_Med2C_cost100, REMIND-MAgPIE 1.7-3.0
69	EMF33_Med2C_full, MESSAGE-GLOBIOM 1.0
70	EMF33_Med2C_full, POLES EMF33
71	EMF33_Med2C_full, REMIND-MAgPIE 1.7-3.0
72	EMF33_Med2C_limbio, MESSAGE-GLOBIOM 1.0
73	EMF33_Med2C_nofuel, MESSAGE-GLOBIOM 1.0
74	EMF33_Med2C_nofuel, POLES EMF33
75	EMF33_Med2C_nofuel, REMIND-MAgPIE 1.7-3.0
76	EMF33_WB2C_cost100, AIM/CGE 2.1
77	EMF33_WB2C_cost100, IMAGE 3.0.2
78	EMF33_WB2C_cost100, MESSAGE-GLOBIOM 1.0
79	EMF33_WB2C_cost100, POLES EMF33
80	EMF33_WB2C_cost100, REMIND-MAgPIE 1.7-3.0
81	EMF33_WB2C_full, IMAGE 3.0.2
82	EMF33_WB2C_full, MESSAGE-GLOBIOM 1.0
83	EMF33_WB2C_full, POLES EMF33
84	EMF33_WB2C_full, REMIND-MAgPIE 1.7-3.0
85	EMF33_WB2C_limbio, MESSAGE-GLOBIOM 1.0
86	EMF33_WB2C_limbio, POLES EMF33
87	EMF33_WB2C_limbio, REMIND-MAgPIE 1.7-3.0
88	EMF33_WB2C_nobeccs, POLES EMF33
89	EMF33_WB2C_nobeccs, REMIND-MAgPIE 1.7-3.0
90	EMF33_WB2C_nofuel, IMAGE 3.0.2
91	EMF33_WB2C_nofuel, MESSAGE-GLOBIOM 1.0
92	EMF33_WB2C_nofuel, POLES EMF33
93	EMF33_WB2C_nofuel, REMIND-MAgPIE 1.7-3.0
94	EMF33_WB2C_none, REMIND-MAgPIE 1.7-3.0
95	EMF33_tax_hi_full, AIM/CGE 2.1
96	EMF33_tax_hi_full, IMAGE 3.0.2
97	EMF33_tax_hi_full, MESSAGE-GLOBIOM 1.0
98	EMF33_tax_hi_full, POLES EMF33
99	EMF33_tax_hi_full, REMIND-MAgPIE 1.7-3.0
100	IMA15-AGInt, IMAGE 3.0.1
101	IMA15-Def, IMAGE 3.0.1
102	IMA15-Eff, IMAGE 3.0.1
103	IMA15-LiStCh, IMAGE 3.0.1

104	IMA15-LoNCO2, IMAGE 3.0.1
105	IMA15-Pop, IMAGE 3.0.1
106	IMA15-RenElec, IMAGE 3.0.1
107	PEP_1p5C_full_NDC, REMIND-MAgPIE 1.7-3.0
108	PEP_1p5C_full_eff, REMIND-MAgPIE 1.7-3.0
109	PEP_1p5C_full_goodpractice, REMIND-MAgPIE 1.7-3.0
110	PEP_1p5C_full_netzero, REMIND-MAgPIE 1.7-3.0
111	PEP_1p5C_red_eff, REMIND-MAgPIE 1.7-3.0
112	PEP_2C_full_NDC, REMIND-MAgPIE 1.7-3.0
113	PEP_2C_full_eff, REMIND-MAgPIE 1.7-3.0
114	PEP_2C_full_goodpractice, REMIND-MAgPIE 1.7-3.0
115	PEP_2C_full_netzero, REMIND-MAgPIE 1.7-3.0
116	PEP_2C_red_NDC, REMIND-MAgPIE 1.7-3.0
117	PEP_2C_red_eff, REMIND-MAgPIE 1.7-3.0
118	PEP_2C_red_goodpractice, REMIND-MAgPIE 1.7-3.0
119	PEP_2C_red_netzero, REMIND-MAgPIE 1.7-3.0
120	SMP_1p5C_Def, REMIND-MAgPIE 1.7-3.0
121	SMP_1p5C_Sust, REMIND-MAgPIE 1.7-3.0
122	SMP_1p5C_early, REMIND-MAgPIE 1.7-3.0
123	SMP_1p5C_lifesty, REMIND-MAgPIE 1.7-3.0
124	SMP_1p5C_regul, REMIND-MAgPIE 1.7-3.0
125	SMP_2C_Def, REMIND-MAgPIE 1.7-3.0
126	SMP_2C_Sust, REMIND-MAgPIE 1.7-3.0
127	SMP_2C_early, REMIND-MAgPIE 1.7-3.0
128	SMP_2C_lifesty, REMIND-MAgPIE 1.7-3.0
129	SMP_2C_regul, REMIND-MAgPIE 1.7-3.0
130	SSP1-19, AIM/CGE 2.0
131	SSP1-19, GCAM 4.2
132	SSP1-19, IMAGE 3.0.1
133	SSP1-19, MESSAGE-GLOBIOM 1.0
134	SSP1-19, REMIND-MAgPIE 1.5
135	SSP1-19, WITCH-GLOBIOM 3.1
136	SSP1-26, GCAM 4.2
137	SSP1-26, IMAGE 3.0.1
138	SSP1-26, MESSAGE-GLOBIOM 1.0
139	SSP1-26, REMIND-MAgPIE 1.5
140	SSP1-26, WITCH-GLOBIOM 3.1

141	SSP1-34, GCAM 4.2
142	SSP1-34, IMAGE 3.0.1
143	SSP1-34, MESSAGE-GLOBIOM 1.0
144	SSP1-34, REMIND-MAgPIE 1.5
145	SSP1-34, WITCH-GLOBIOM 3.1
146	SSP2-19, AIM/CGE 2.0
147	SSP2-19, GCAM 4.2
148	SSP2-19, MESSAGE-GLOBIOM 1.0
149	SSP2-19, REMIND-MAgPIE 1.5
150	SSP2-26, AIM/CGE 2.0
151	SSP2-26, GCAM 4.2
152	SSP2-26, IMAGE 3.0.1
153	SSP2-26, MESSAGE-GLOBIOM 1.0
154	SSP2-26, REMIND-MAgPIE 1.5
155	SSP2-26, WITCH-GLOBIOM 3.1
156	SSP2-34, GCAM 4.2
157	SSP2-34, MESSAGE-GLOBIOM 1.0
158	SSP2-34, REMIND-MAgPIE 1.5
159	SSP3-34, AIM/CGE 2.0
160	SSP3-34, MESSAGE-GLOBIOM 1.0
161	SSP4-19, WITCH-GLOBIOM 3.1
162	SSP4-26, AIM/CGE 2.0
163	SSP4-26, GCAM 4.2
164	SSP4-26, IMAGE 3.0.1
165	SSP4-26, WITCH-GLOBIOM 3.1
166	SSP4-34, GCAM 4.2
167	SSP5-19, GCAM 4.2
168	SSP5-19, REMIND-MAgPIE 1.5
169	SSP5-26, AIM/CGE 2.0
170	SSP5-26, GCAM 4.2
171	SSP5-26, REMIND-MAgPIE 1.5
172	SSP5-34, GCAM 4.2
173	SSP5-34, REMIND-MAgPIE 1.5
174	TERL_15D_LowCarbonTransportPolicy, AIM/CGE 2.1
175	TERL_15D_NoTransportPolicy, AIM/CGE 2.1
176	TERL_2D_LowCarbonTransportPolicy, AIM/CGE 2.1
177	TERL_2D_NoTransportPolicy, AIM/CGE 2.1

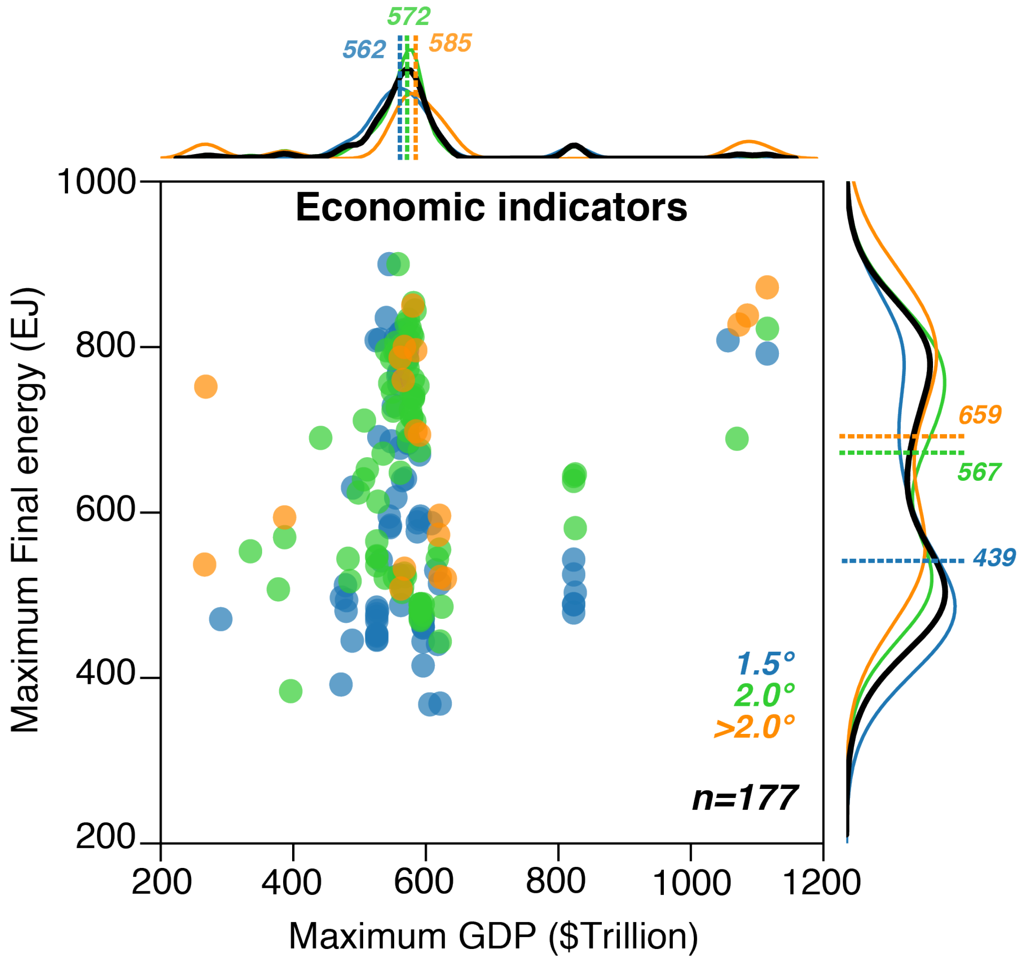

Supplementary Figure 1 | Economic indicators for net-zero scenarios. Maximum final energy is plotted against maximum GDP ultimately reached in net-zero scenarios. Points represent individual scenarios, with color corresponding to warming level (blue = <1.5˚C, green = 2.0˚C, orange = >2.0˚C) and probability density distributions shown along each axis for each warming level (colors corresponding to warming levels). Median lines for each warming group are shown as dashed lines along distributions.

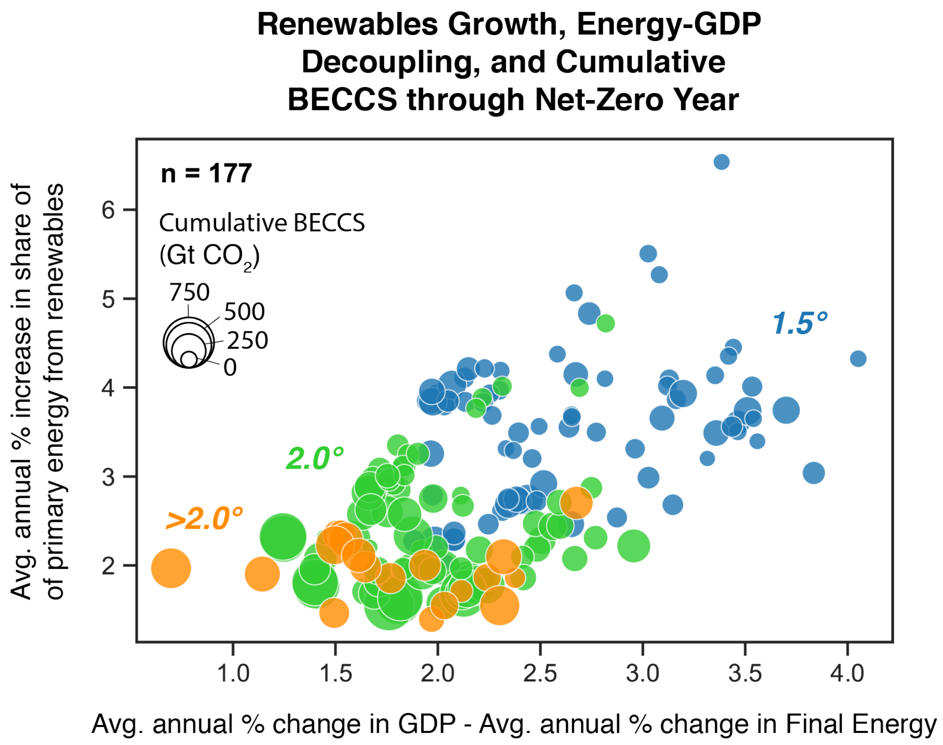

Supplementary Figure 2 | Rate of energy system transformation by warming group up to net-zero year. Rate of energy system transformation is represented by the difference between average annual % change in GDP and final energy (x-axis) and the average annual % change in renewable energy share of primary energy (y-axis) through each scenario's net-zero year. Colors of points indicate warming level (blue = <1.5˚C, green = 2.0˚C, orange = >2.0˚C), and size of points corresponds to the cumulative carbon sequestered through bioenergy with carbon capture and storage (BECCS) through the net-zero year.

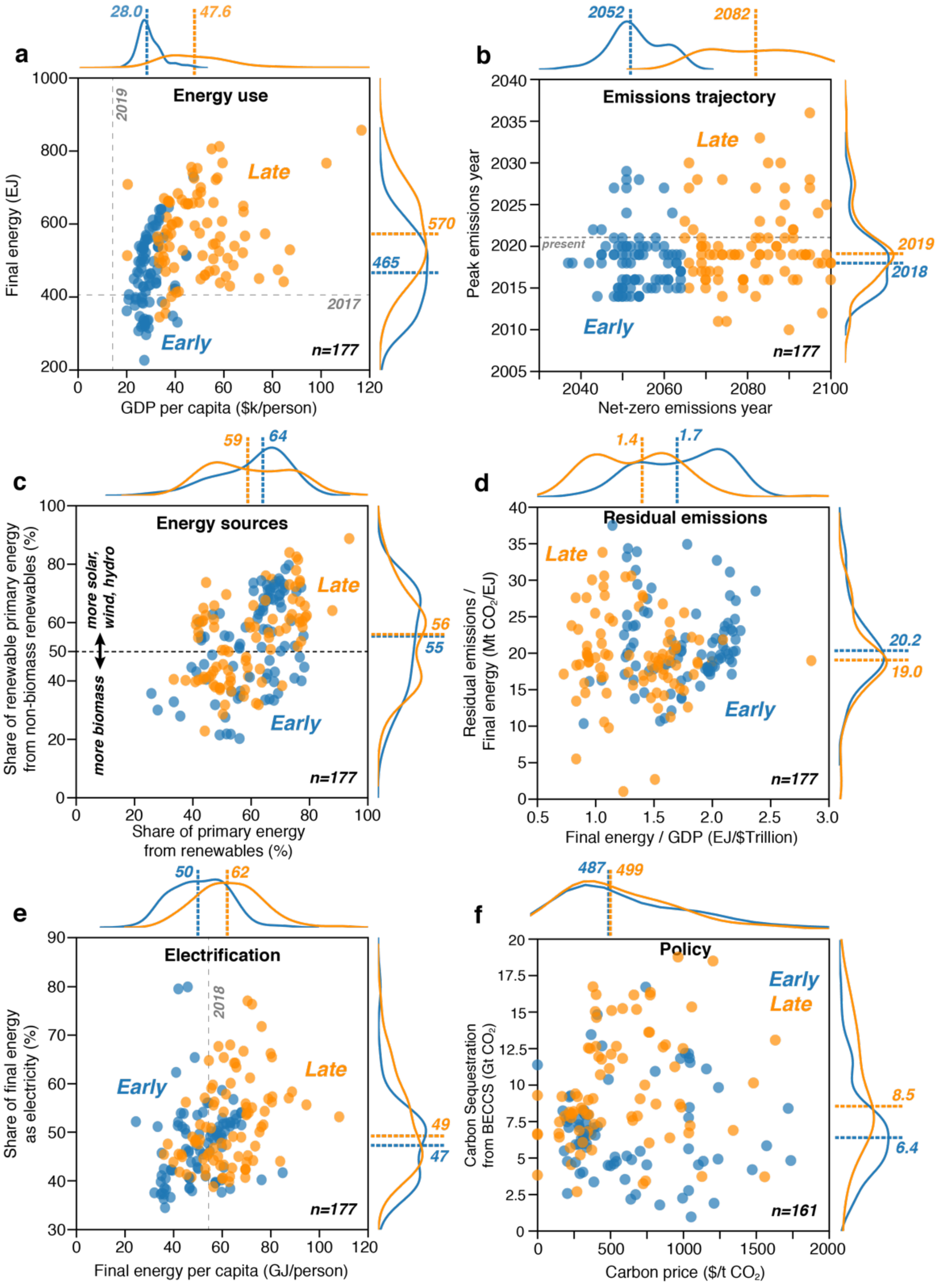

Supplementary Figure 3 | Early vs. Late net-zero scenarios. Global scenarios that reach net-zero emissions before or at the median net-zero year 2064 ("Early") vs. after the median net-zero year ("Late") show differences in energy use (**a**), emissions trajectory (**b**), energy sources (**c**), residual emissions (**d**), electrification (**e**), and policy (**f**). Points represent individual scenarios, with frequency of scenarios shown along each axis for Early and Late scenario groups. Colored dashed lines and values indicate medians for Early vs. Late scenario groups. Gray dashed lines indicate reference values for the year shown in gray.

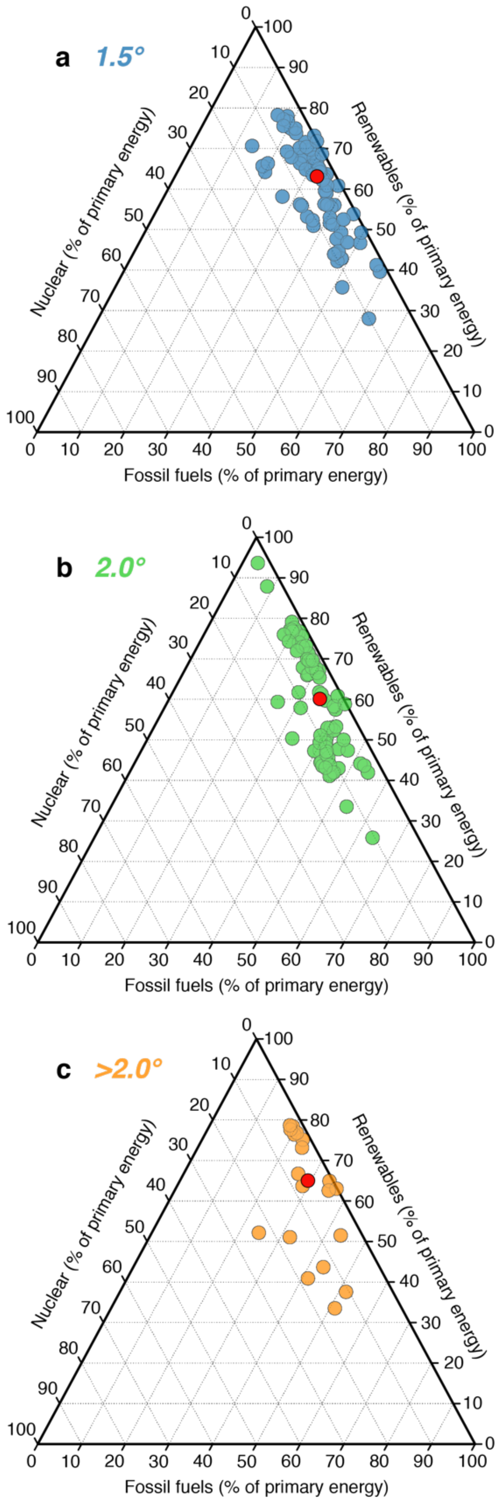

Supplementary Figure 4 | Primary Energy Sources in Global Net-Zero Emissions Scenarios. Ternary diagrams show the percentage of primary energy from renewables (right axis), fossil fuels (bottom axis), and nuclear (left axis) for <1.5˚C (**a**), 2.0˚C (**b**), and >2.0˚C (**c**) scenarios. The three axis values for each individual point sum to 100%. Red points indicate the geometric median for each respective warming group.

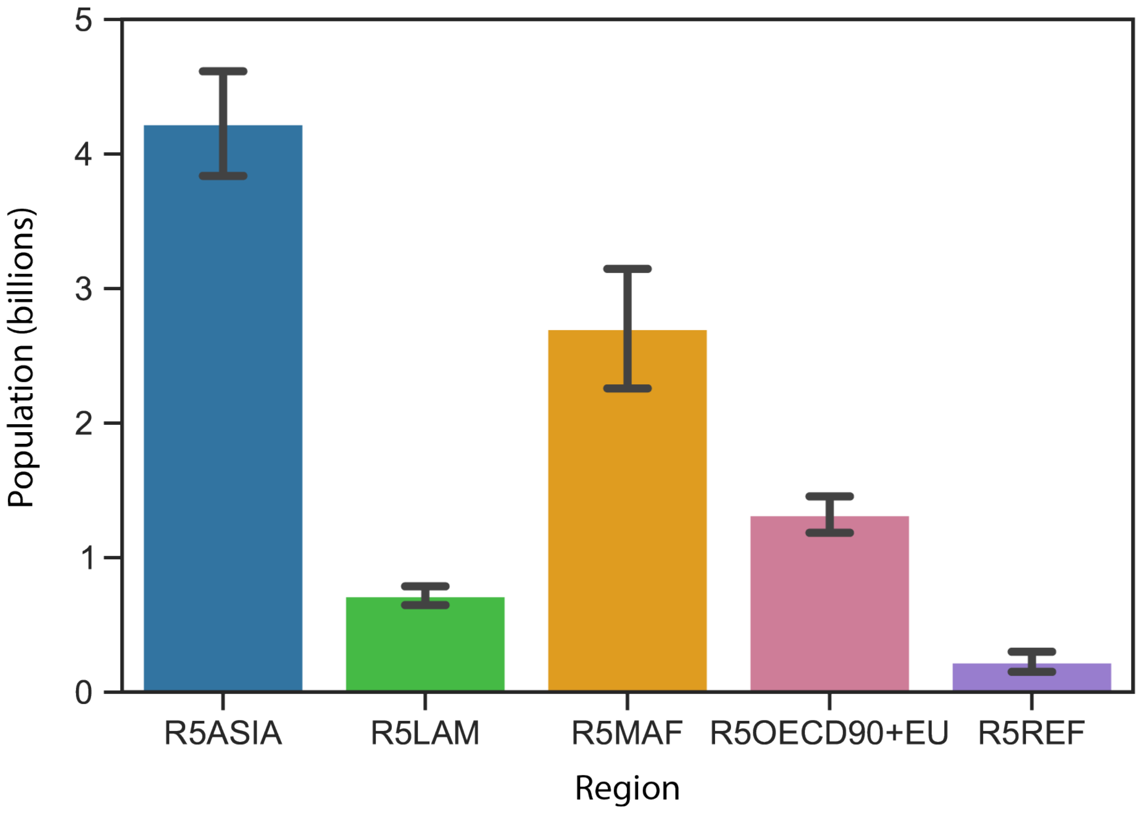

Supplementary Figure 5 | Regional populations in net-zero year. Population in scenario net-zero years for R5ASIA (Asia), R5LAM (Latin America), R5MAF (Middle East+Africa), R5OECD90+EU (OECD and EU countries), and R5REF (Eastern Europe+Russia). Error bars indicate 1 standard deviation.

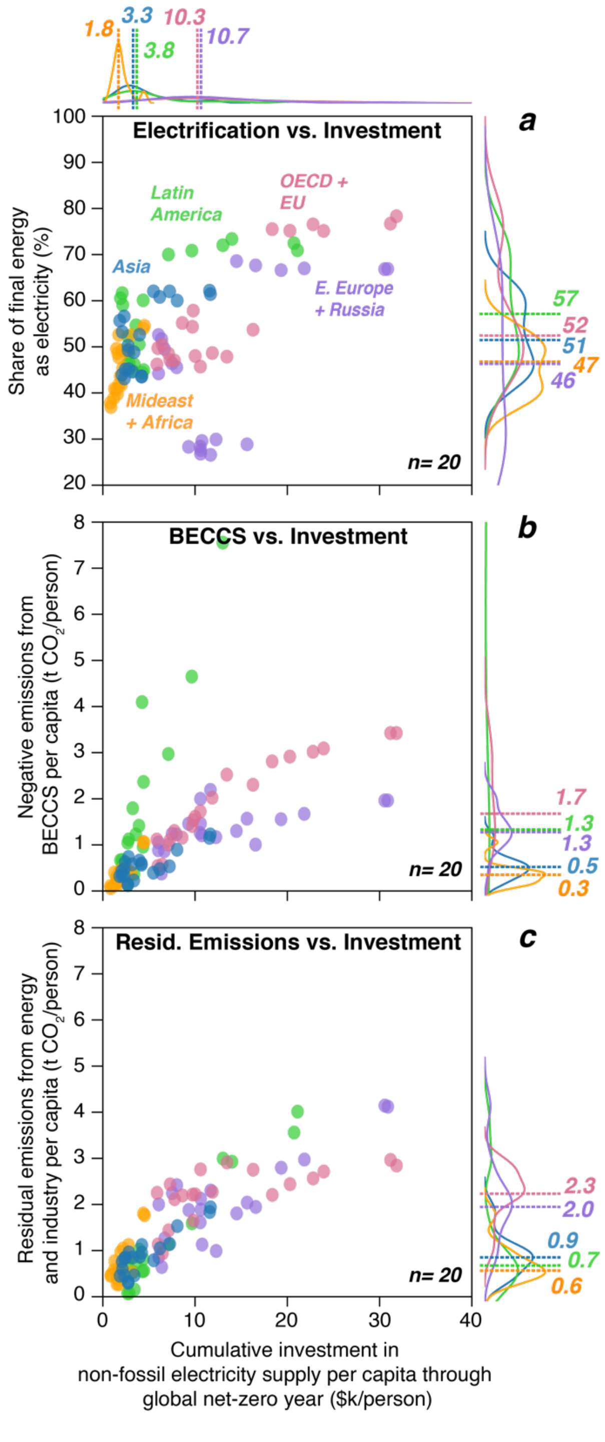

Supplementary Figure 6 | Regional investment vs. electrification, negative emissions from BECCS, and residual energy+industry emissions. Scenarios that reach net-zero emissions globally and have regional outputs for investment in non-fossil electricity supply (n=20 scenarios with all regions, for a total of 100 data points) show regional differences in share of final energy as electricity (**a**), per capita negative emissions from BECCS (**b**), and per capita residual emissions from energy and industry (**c**). Points represent individual scenarios, with frequency of scenarios shown along each axis for each region (Asia = blue, Latin America = green, Middle East+Africa = orange, OECD+EU countries = pink, and Eastern Europe+Russia = purple). Colored dashed lines and values indicate medians for each region.

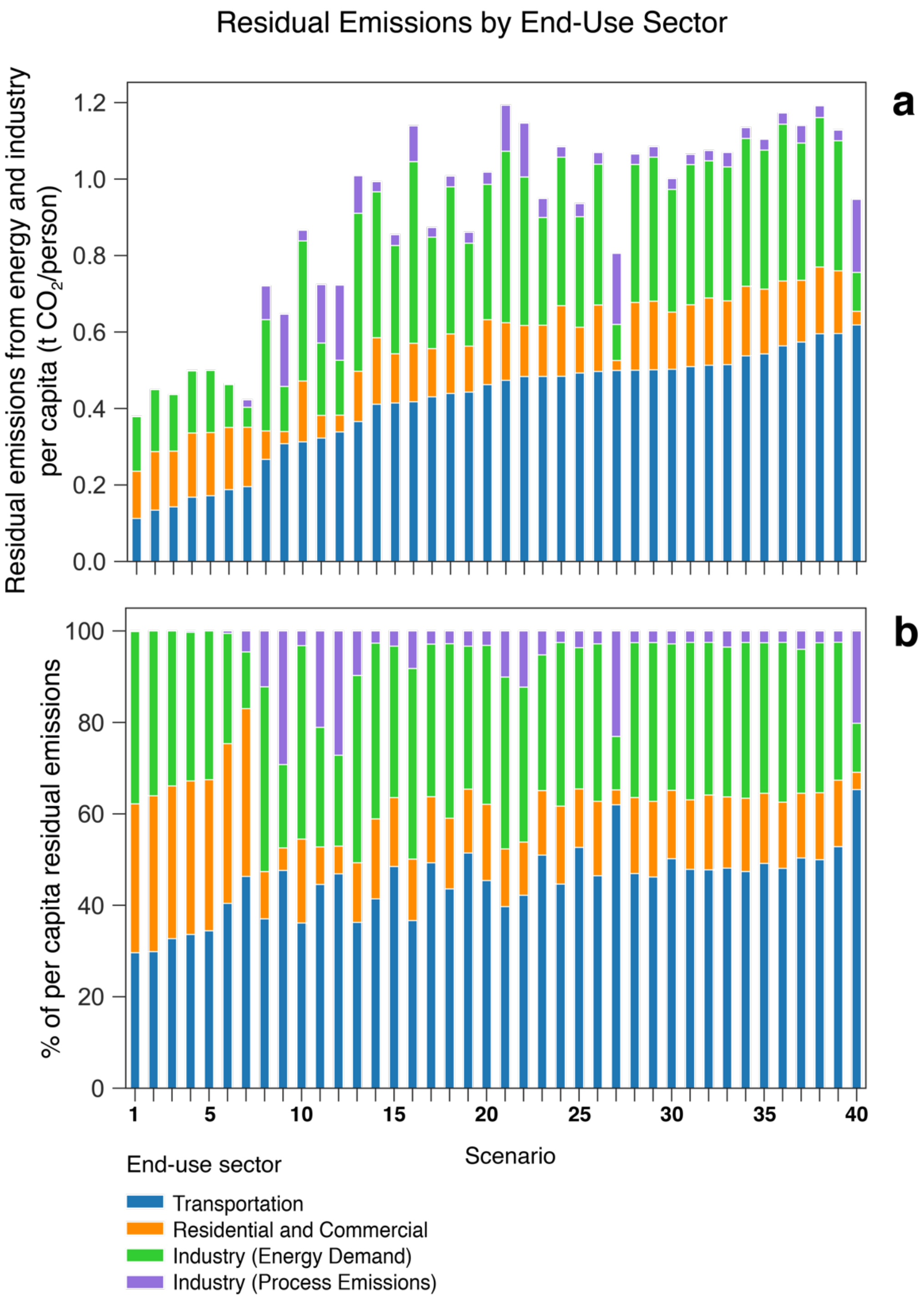

Supplementary Figure 7 | Residual emissions by end-use sector in global net-zero year. Each bar represents an individual scenario output, numbered 1-40. Please see Table S2 for corresponding list of scenarios and numbers for this figure. Panel (**a**) shows total per capita residual emissions from four end-use sectors as the height of each bar, and the amount from each end-use sector is represented by color: transportation = blue, residential and commercial = orange, industry (energy demand) = green, and industry (process emissions) = purple. Panel (**b**) shows each end-use sector as a percentage of the total residual emissions from the four sectors.

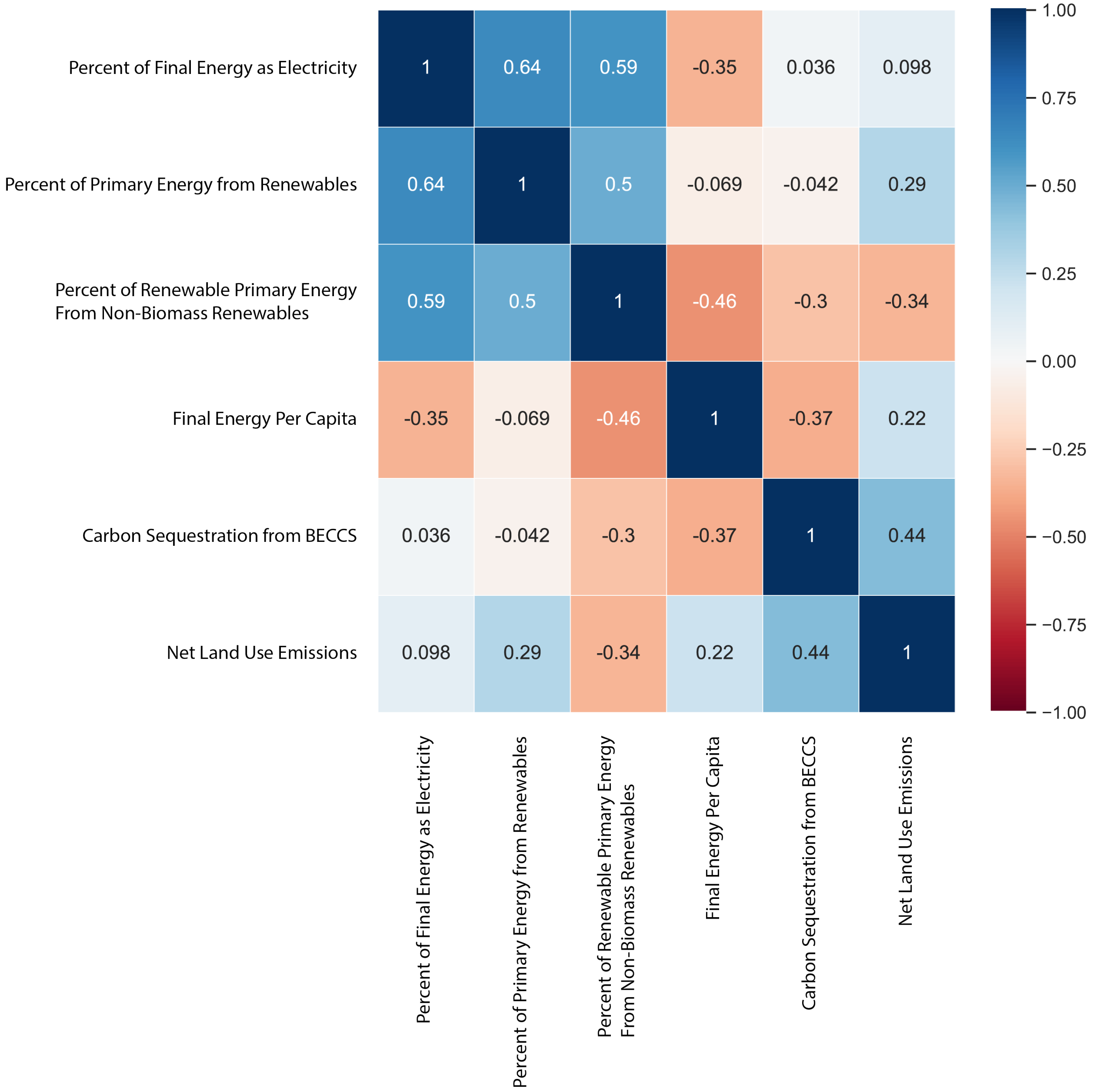

Supplementary Figure 8 | Pairwise Correlation Matrix for Figure 4 Parameters. Pairwise correlation coefficients for Figure 4 columns are shown in a matrix, with positive correlation coefficients shaded blue and negative correlation coefficients shaded red. Darker shades indicate stronger positive or negative correlations.

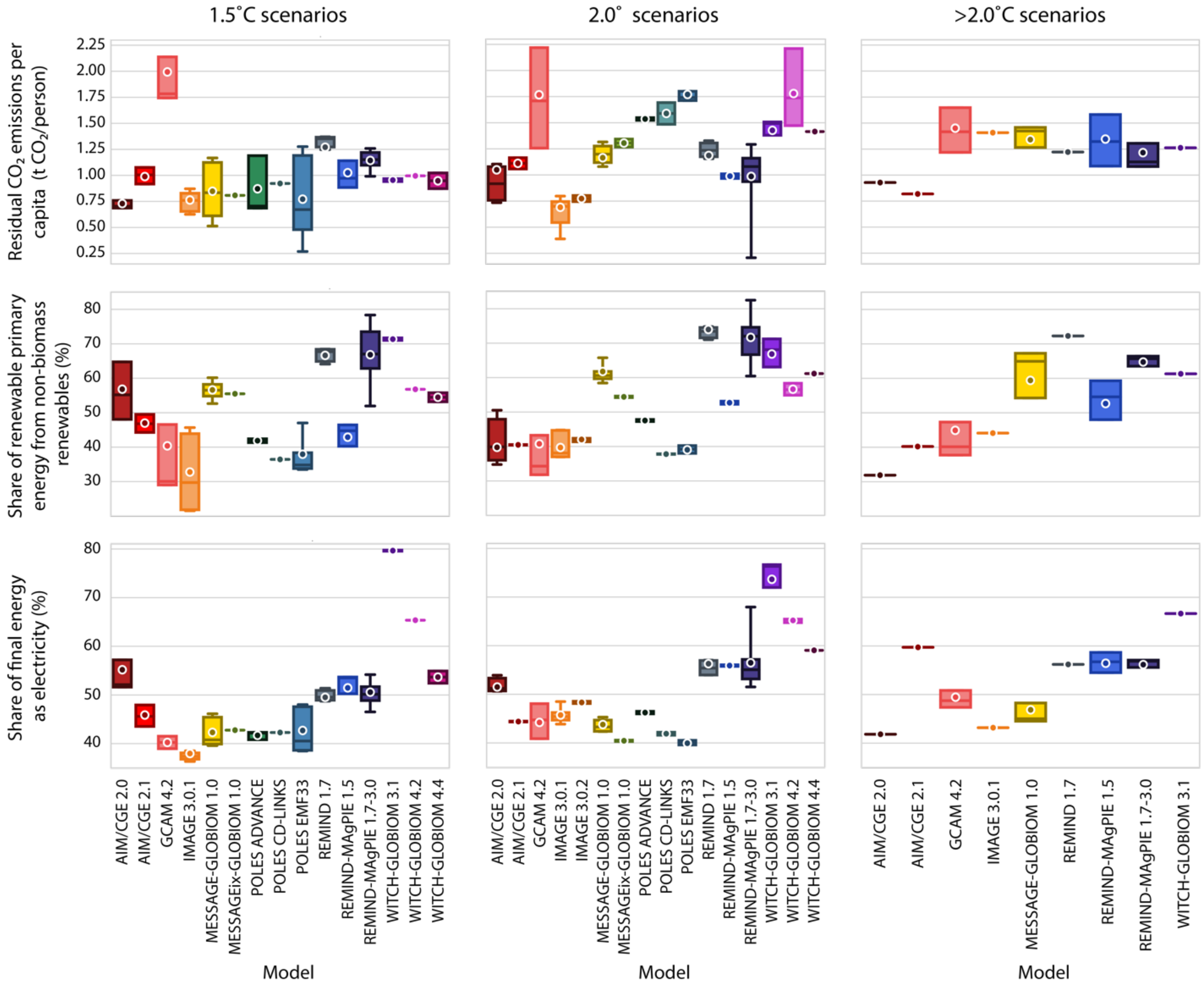

Supplementary Figure 9 | Model comparison for parameters in global net-zero year. Residual CO_2 emissions per capita (top row), the share of renewable primary energy from non-biomass sources (middle row), and the share of final energy as electricity (bottom row) are shown for 1.5˚C (left column), 2.0˚C (middle column), and >2.0˚C (right column) scenarios, broken out by model. In each case, the boxes show the range from 25th to 75th percentiles, and the whiskers indicate the 5th and 95th percentiles where applicable (some models did not have enough scenario points to show 5th and 95th percentiles, since any points outside the box range were outliers). Lines and circles within the boxes denote the median and mean values, respectively. 15 models ran 1.5˚ scenarios, 16 models ran 2.0˚C scenarios, and 9 models ran >2.0˚C scenarios that reach net-zero emissions. For ease of comparison, box colors are consistent for each model throughout the figure (each model is always the same color across plots).

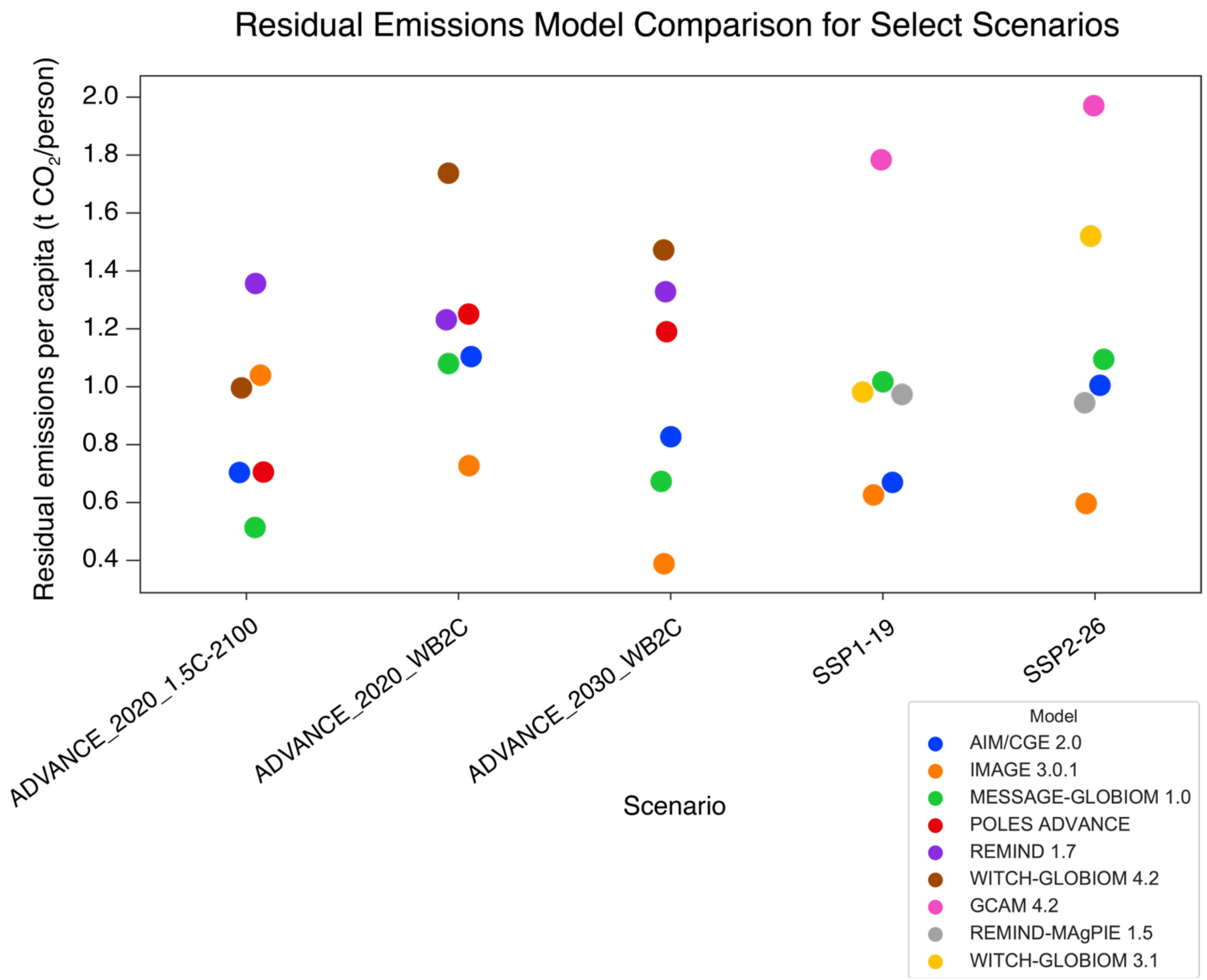

Supplementary Figure 10 | Residual emissions model/scenario comparison in global net-zero year. Per capita residual emissions from energy+industry are shown for five scenarios, each of which was run by 6 models. Models AIM/CGE 2.0 (blue), IMAGE 3.0.1 (orange), and MESSAGE-GLOBIOM 1.0 (green) ran all five scenarios; Models POLES ADVANCE (red), REMIND 1.7 (purple), and WITCH-GLOBIOM 4.2 (brown) ran scenarios ADVANCE_2020_1.5C-2100, ADVANCE_2020_WB2C, and ADVANCE_2030_WB2C; Models GCAM 4.2 (pink), REMIND-MAgPIE 1.5 (gray), and WITCH-GLOBIOM 3.1 (yellow) ran scenarios SSP1-19 and SSP2-26. Of the models that ran all five scenarios, IMAGE 3.0.1 has the lowest residual emissions value for four of the five scenarios, AIM/CGE 2.0 is the bottom half of model values for all five scenarios, and MESSAGE-GLOBIOM 1.0 is in the bottom half of model values for four of the five scenarios. Of the models that only ran the ADVANCE scenarios, WITCH-GLOBIOM 4.2 is the highest in two of the three ADVANCE scenarios and is in the top half of model values for ADVANCE_2020_1.5C-2100, while POLES ADVANCE and REMIND 1.7 are in the top half of model values for two of the three ADVANCE scenarios, and REMIND 1.7 is the highest for ADVANCE_2020_1.5C-2100. Of the models that only ran the SSP scenarios, GCAM 4.2 is by far the highest for both SSP1-19 and SSP2-26, while REMIND-MAgPIE 1.5 in the bottom half of models for both and WITCH-GLOBIOM 3.1 is in the top half of models for both.

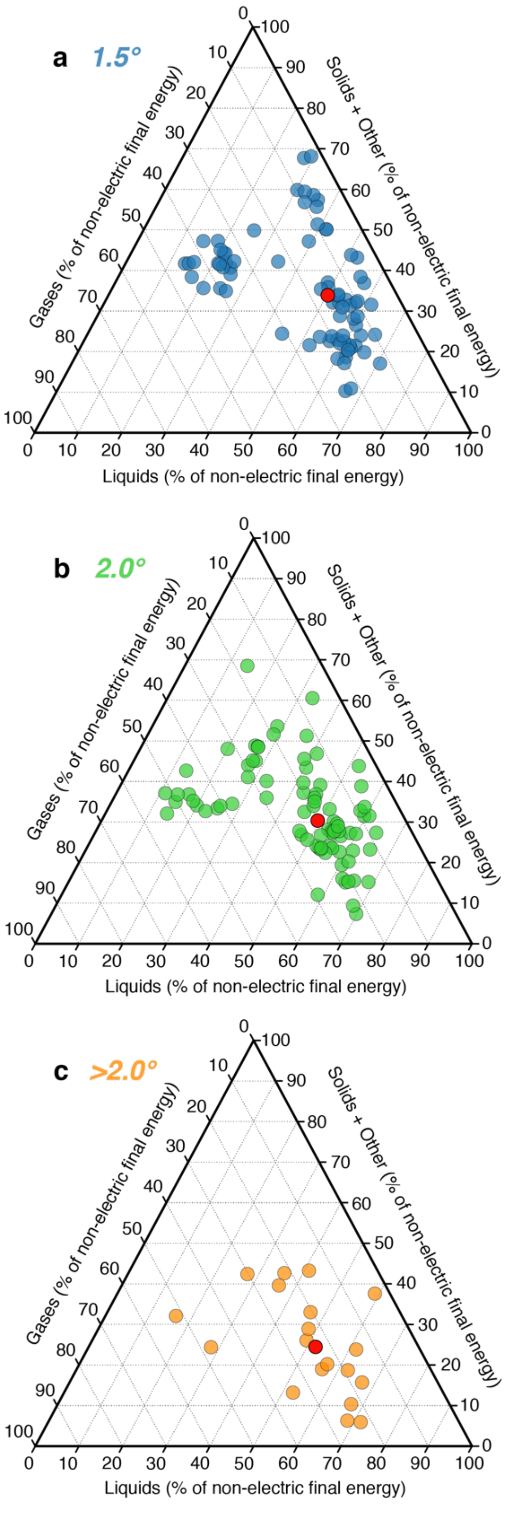

Supplementary Figure 11 | Fuel Types for Non-Electric Final Energy in Global Net-Zero Emissions Scenarios. Ternary diagrams show the percentage of non-electric final energy from gases (left axis), liquids (bottom axis), and solids + other (right axis) for <1.5˚C (**a**), 2.0˚C (**b**), and >2.0˚C (**c**) scenarios. The three axis values for each individual point sum to 100%. Red points indicate the geometric median for each respective warming group.

Supplementary Table 2 | List of 40 scenarios evaluated by end-use sector residual emissions in Supplementary Figure 7. Only these 40 scenarios out of the original 177 had all required output variables for end-use sector analysis. Scenarios are listed first, followed by the model that ran the scenario (format: scenario, model).

1	IMA15-RenElec, IMAGE 3.0.1
2	IMA15-LiStCh, IMAGE 3.0.1
3	IMA15-LoNCO2, IMAGE 3.0.1
4	IMA15-Pop, IMAGE 3.0.1
5	IMA15-AGInt, IMAGE 3.0.1
6	IMA15-Def, IMAGE 3.0.1
7	IMA15-Eff, IMAGE 3.0.1
8	CEMICS-2.0-CDR8, REMIND 1.7
9	TERL_15D_LowCarbonTransportPolicy, AIM/CGE 2.1
10	SMP_2C_Sust, REMIND-MAgPIE 1.7-3.0
11	CEMICS-1.5-CDR8, REMIND 1.7
12	TERL_2D_LowCarbonTransportPolicy, AIM/CGE 2.1
13	CEMICS-2.0-CDR12, REMIND 1.7
14	SMP_2C_early, REMIND-MAgPIE 1.7-3.0
15	PEP_2C_red_netzero, REMIND-MAgPIE 1.7-3.0
16	CEMICS-2.0-CDR20, REMIND 1.7
17	PEP_2C_red_eff, REMIND-MAgPIE 1.7-3.0
18	SMP_2C_lifesty, REMIND-MAgPIE 1.7-3.0
19	PEP_2C_red_goodpractice, REMIND-MAgPIE 1.7-3.0
20	PEP_1p5C_full_netzero, REMIND-MAgPIE 1.7-3.0
21	CEMICS-1.5-CDR20, REMIND 1.7
22	CEMICS-1.5-CDR12, REMIND 1.7
23	PEP_1p5C_red_eff, REMIND-MAgPIE 1.7-3.0
24	PEP_2C_full_netzero, REMIND-MAgPIE 1.7-3.0
25	PEP_2C_red_NDC, REMIND-MAgPIE 1.7-3.0
26	PEP_1p5C_full_eff, REMIND-MAgPIE 1.7-3.0
27	TERL_15D_NoTransportPolicy, AIM/CGE 2.1
28	SMP_2C_Def, REMIND-MAgPIE 1.7-3.0
29	PEP_2C_full_goodpractice, REMIND-MAgPIE 1.7-3.0
30	SMP_1p5C_early, REMIND-MAgPIE 1.7-3.0
31	SMP_2C_regul, REMIND-MAgPIE 1.7-3.0
32	PEP_2C_full_eff, REMIND-MAgPIE 1.7-3.0
33	PEP_1p5C_full_goodpractice, REMIND-MAgPIE 1.7-3.0

34	PEP_2C_full_NDC, REMIND-MAgPIE 1.7-3.0
35	SMP_1p5C_Def, REMIND-MAgPIE 1.7-3.0
36	SMP_1p5C_lifesty, REMIND-MAgPIE 1.7-3.0
37	PEP_1p5C_full_NDC, REMIND-MAgPIE 1.7-3.0
38	SMP_1p5C_Sust, REMIND-MAgPIE 1.7-3.0
39	SMP_1p5C_regul, REMIND-MAgPIE 1.7-3.0
40	TERL_2D_NoTransportPolicy, AIM/CGE 2.1

Supplementary Table 3 | List of 20 scenarios evaluated by investment in non-fossil electricity supply in Supplementary Figure 6. Only these 20 scenarios (5 regions for each scenario, for a total of 100 data points in Supplementary Fig. 6) out of the original 175 regional scenarios had non-zero regional outputs for investment in non-fossil electricity supply in the global net-zero year. Scenarios are listed first, followed by the model that ran the scenario.

1	ADVANCE_2020_1.5C-2100, AIM/CGE 2.0
2	ADVANCE_2020_1.5C-2100, MESSAGE-GLOBIOM 1.0
3	ADVANCE_2020_1.5C-2100, WITCH-GLOBIOM 4.2
4	ADVANCE_2020_Med2C, MESSAGE-GLOBIOM 1.0
5	ADVANCE_2020_Med2C, WITCH-GLOBIOM 4.2
6	ADVANCE_2020_WB2C, AIM/CGE 2.0
7	ADVANCE_2020_WB2C, MESSAGE-GLOBIOM 1.0
8	ADVANCE_2020_WB2C, WITCH-GLOBIOM 4.2
9	ADVANCE_2030_Med2C, MESSAGE-GLOBIOM 1.0
10	ADVANCE_2030_Med2C, WITCH-GLOBIOM 4.2
11	ADVANCE_2030_Price1.5C, AIM/CGE 2.0
12	ADVANCE_2030_Price1.5C, MESSAGE-GLOBIOM 1.0
13	ADVANCE_2030_Price1.5C, WITCH-GLOBIOM 4.2
14	ADVANCE_2030_WB2C, AIM/CGE 2.0
15	ADVANCE_2030_WB2C, MESSAGE-GLOBIOM 1.0
16	ADVANCE_2030_WB2C, WITCH-GLOBIOM 4.2
17	TERL_15D_LowCarbonTransportPolicy, AIM/CGE 2.1
18	TERL_15D_NoTransportPolicy, AIM/CGE 2.1
19	TERL_2D_LowCarbonTransportPolicy, AIM/CGE 2.1
20	TERL_2D_NoTransportPolicy, AIM/CGE 2.1
